# Women with Epilepsy: Anti-epileptic Drugs and Perinatal Outcomes

**DOI:** 10.7759/cureus.5642

**Published:** 2019-09-13

**Authors:** Tooba Kashif, Nida Fathima, Norina Usman, Aisha Qaseem, Joseph S Jayaraj

**Affiliations:** 1 Internal Medicine, California Institute of Behavioral Neurosciences and Psychology, Fairfield, USA; 2 Internal Medicine, Veterans Affairs Palo Alto Health Care System - Stanford University School of Medicine, Palo Alto, USA

**Keywords:** pregnancy complications, anti-epileptic drugs, neuro-cognitive, malformation, congenital malformations, perinatal outcomes, pregnant women

## Abstract

Epilepsy is a chronic neurological condition that requires treatment throughout the pregnancy. Seizures should be well controlled before conception with a specific type of anti-epileptic drug (AED) for each epileptic syndrome. The selection of AED is crucial in women with epilepsy (WWE). AEDs with the lowest malformations rates should be used for treatment during pregnancy. Valproate should be avoided in WWE of childbearing age as it is associated with the highest risk of neurocognitive malformations. However, pregnancy might alter the levels of AEDs, which can lead to an increase in seizure frequency. It is important to monitor AED levels and make necessary dose adjustments to control seizures during pregnancy. WWE should be treated with the lowest possible dose allowed and preferably with a single AED to avoid harmful effects on the developing fetus. Women should be counseled to take folic acid during pregnancy as it reduces the risks for cardiovascular, genitourinary, and neural tube defects. Generally, WWE usually have normal pregnancies and can bear healthy offspring. Pregnant women need continuous follow-up in a coordinated manner with the neurologist and obstetrician to assess for adverse pregnancy and fetal outcomes.

## Introduction and background

Epilepsy is a chronic disorder that affects the general population, with an annual incidence rate of 40-70 per 100,000 persons per year [1]. It is a neurological condition characterized by an increased risk of recurrent, unprovoked seizures and is managed by either lifelong intake of different classes of anti-epileptic drugs (AEDs) or surgery. Even with advancements in the surgical treatment for epilepsy, AEDs are widely used for the treatment of different types of epileptic syndrome. In the USA, approximately 0.3-0.5% of all childbirths are in women with epilepsy (WWE) [2]. The optimal management for WWE during pregnancy presents a challenging situation for clinicians to balance the benefits of treatment with achieving an adequate level of AEDs for seizure control versus minimal exposure to the developing fetus to prevent harm.

In this literature review, we will discuss the impact of physiological changes during pregnancy, changes in AED levels, alterations in seizure frequency, the malformation risks of different AEDs, the effect of epilepsy on the general health of WWE, the optimal management of epilepsy, and which drugs are considered safe during pregnancy. More research is needed in the area of new AEDs such as oxcarbazepine, eslicarbazepine, and clobazam and their effects on pregnancy outcomes in large population groups.

## Review

^AED levels and clearance during pregnancy^

Epilepsy is a condition that should be well controlled before conception by making necessary dose adjustments and selecting the optimal therapy for patients. WWE might experience an increase in seizure frequency during pregnancy due to an alteration in AED levels, which could be due to many factors such as decreased drug levels due to an increase in plasma volume, increased hepatic clearance, hormonal changes leading to an increase in the metabolism of drugs, and other psychological changes during pregnancy. It is essential to monitor AED levels during pregnancy and in the postpartum period and to adjust the dosage of medications to achieve adequate seizure control [3]. A retrospective analysis was conducted by Reisinger et al. at Emory Epilepsy Center to study the effect of multiple AED clearance changes and its association with seizure frequency. This analysis was performed in 115 pregnancies on 95 WWE, and AED blood levels (ABLs) were measured routinely during pregnancy and used to calculate clearance of AEDs several times in pregnancy. Data were analyzed to associate the changes in clearance, dosage during pregnancy, and the association between ABLs and changes in seizure frequency. Lamotrigine and levetiracetam reflected significant changes in clearance. A total of 38.4% of women experienced an increase in seizure frequency despite increasing the dose of AEDs. The most important finding of the study, i.e., seizure deterioration, was noted to be more likely to be observed in women who had seizures in a year before conception (*p* < 0.001) and women with localized epilepsy (*p* = 0.05). When the ABL decreases more than 35% from the preconception baseline, it can lead to an increase in seizure frequency. Significant monitoring of serum AED levels and dose adjustments are required to achieve seizure control in pregnant WWE [4].

^WWE and associated pregnancy and perinatal risk factors^

A population-based cohort study was conducted in Sweden by Razaz et al. including WWE with exposure to AEDs and non-exposed groups to study the association between pregnancy and perinatal complications in both cohorts from 1997 to 2011. Pregnancy, delivery, and perinatal outcomes were calculated using multivariate Poisson log-linear regression adjusted risk ratio (aRR) and other confounders. A total of 1,429,652 births were included in the study, with 5,373 pregnancies in WWE. Compared with non-epileptic women, epileptic women were at an increased risk of adverse pregnancy and delivery outcomes: preeclampsia (aRR = 1.24), infection (aRR = 1.85), placental abruption (aRR = 1.68), induction of labor (aRR = 1.31), elective cesarean section (aRR = 1.58), and emergency cesarean section (aRR = 1.09) (Table 1). Infants of WWE are also at increased risks of stillbirths (aRR = 1.55), small for gestational age (aRR = 1.25), congenital malformation (aRR = 1.48), major malformations (aRR = 1.61), asphyxia-related complications (aRR = 1.75), and respiratory distress syndrome (aRR = 1.49) compared with infants of women without epilepsy (WOE) (Table 2). However, intake of AEDs does not seems to increase the risk of pregnancy and perinatal complications, except for the increased rate of induction of labor (aRR = 1.30). In this study, lamotrigine and carbamazepine are the two most commonly used AEDs in 77% of patients [5]. 

**Table 1:** Women with Epilepsy and Pregnancy Outcomes CI: confidence interval.

Adverse Pregnancy Complications	Adjusted Risk Ratio (95 % CI)
Preeclampsia	1.24 (1.07-1.43)
Maternal Infection	1.85 (1.43-2.29)
Placental Abruption	1.68 (1.18-2.38)
Prolonged Labor	0.86 (0.62-1.18)
Induction of Labor	1.31 (1.21-1.40)
Elective Cesarean Section	1.58 (1.45-1.71)
Emergency Cesarean Section	1.09 (1.00-1.20)
Postpartum Hemorrhage	1.11 (0.97-1.26)

**Table 2:** Women with Epilepsy and Perinatal Outcomes

Perinatal Outcomes	Adjusted Risk Ratio
Stillbirth	1.55 (1.05-2.30)
Medically Indicated Preterm Birth	1.24 (1.08-1.43)
Spontaneous Preterm Birth	1.34 (1.20-1.53)
Small for Gestational Age	1.25 (1.13-1.30)
Neonatal Infections	1.42 (1.17-1.73)
Congenital Malformations	1.48 (1.35-1.62)
Major Malformations	1.61 (1.43-1.61)
Asphyxia-Related Complications	1.75 (1.26-2.42)
Apgar Score of 0-3 at 5 Minutes	2.42 (1.62-3.61)
Neonatal Hypoglycemia	1.53 (1.34-1.75)
Neonatal Respiratory Distress	1.48 (1.30-1.68)

Another retrospective cohort study was conducted by Macdonald et al. that used USA hospitalization records from 2007 to 2011 that includes 69,385 WWE and 20,449,532 WOE. One of the findings of the survey is that the death rate is significantly higher among WWE during delivery: 80 per 100,000 as compared to 6 per 100,000 in WOE (adjusted odds ratio [OR]: 11.46 [95% confidence interval, CI: 8.64-15.19]). Also, WWE are at an increased risk of other adverse pregnancy outcomes that includes preeclampsia (adjusted OR: 1.59 [95% CI: 1.54-1.63]), preterm labor (adjusted OR: 1.54 [95% CI: 1.50-1.57]), and stillbirth (adjusted OR: 1.27 [95% CI: 1.17-1.38]), and an increased risk of cesarean section (adjusted OR: 1.40 [95% CI: 1.38-1.42]) [6]. Epilepsy increases the risk during pregnancy and birth and hence needs continuous monitoring of women in a coordinated fashion.

^Sudden unexpected deaths and mortality in WWE^

WWE have a tenfold increase in mortality risk as compared to WOE, but the absolute risk is low and only about 0.1% [6]. Most WWE have a normal pregnancy and give birth to normal healthy offspring [7]. Edey et al. analyzed the 2011 report of the UK confidential death inquiries into maternal death and epilepsy rates in pregnancy. The report included 2,291,493 maternities between 2006 and 2008, with 0.6% of all pregnancies in WWE. Fourteen deaths were related to epilepsy, of which 11 were due to sudden terminal epileptic seizures (SUDEP). Nine women who died were on lamotrigine therapy. The lamotrigine level decreases significantly during pregnancy and may lead to an uncontrolled seizure, but, simply, it could reflect the prescribing practice of AEDs in the United Kingdom. It is important to maintain drug levels during pregnancy and the postpartum period as well to achieve steady seizure control [8].

^Malformation risks of AEDs^

Epilepsy requires continuous maternal treatment during the gestational and postpartum periods to achieve adequate seizure control with minimal possible exposure to the fetus as it might lead to major congenital malformations (MCMs) and affect cognitive development. A longitudinal prospective cohort study based on the EURAP (International Registry of Antiepileptic Drugs and Pregnancy) was conducted to study the association between teratogenicity and different AEDs with different dosages. A total of 7,555 pregnancies between June 1999 and May 2016 were included in the study. Rate of MCMs was 10.3% for valproate, 6.5% for phenobarbital, 6.4% for phenytoin, 5.5% for carbamazepine, 3.9% for topiramate, 3.0% for oxcarbazepine, 2.9% for lamotrigine, and 2.8% for levetiracetam (Table 3). Risks of MCMs increase with the dosing of medications at the time of conception with phenobarbital, carbamazepine, lamotrigine, and valproate. Carbamazepine and valproate showed higher risks of MCMs for all doses. Valproate at a dose of 650 mg/day or less can cause an increased risk of MCM compared with carbamazepine at a dose of more than 700 mg/day. Higher risks of malformations with levetiracetam was seen with doses of 250-4,000 mg/day and oxcarbazepine with doses of 75-4,500 mg/day [9]. Different AEDs can cause different types of malformation in the developing system. AEDs that are folic acid antagonists as well, such as phenytoin, phenobarbital, carbamazepine, and primidone, increase the risk of neural tube, orofacial, cardiovascular, and urinary tract defects, which can be minimized by optimal use of a folic acid component of multivitamins [10]. The North American Antiepileptic Drug Pregnancy Registry (NAAPR) that enrolled more than 3,000 WWE and on AEDs has reported malformation rates of monotherapy for valproate to be highest 9.3%, followed by phenobarbital (5.5%), topiramate (4.2%), carbamazepine (3%), phenytoin (2.9%), levetiracetam (2.4%), and lamotrigine (2.0%) (Table 4) [11]. The Epilepsy Therapy Project founded the Health Outcomes in Pregnancy and Epilepsy (HOPE) forum with nine international groups that also outlines current knowledge and determines paths for future research for WWE to understand the impact of AEDs on the fetus and other implications. [12]. Several AED pregnancy registries have been developed to guide clinicians and obstetricians on the management of epilepsy.

**Table 3:** EURAP International Registry and MCM Rates EURAP: International Registry of Antiepileptic Drugs and Pregnancy; MCM, major congenital malformation; AED, anti-epileptic drug.

AED	MCM Rates
Valproate	10.3%
Phenobarbital	6.5%
Phenytoin	6.4%
Carbamazepine	5.5%
Topiramate	3.9%
Oxcarbazepine	3.0%
Lamotrigine	2.9%
Levetiracetam	2.8%

**Table 4:** NAAPR and MCM Rates with AEDs NAAPR, North American Anti-Epileptic Drug Pregnancy Registry; MCM, major congenital malformation; AED, anti-epileptic drug.

AED	MCM Rates
Valproate	9.3%
Phenobarbital	5.5%
Topiramate	4.2%
Carbamazepine	3%
Phenytoin	2.9%
Levetiracetam	2.4%
Lamotrigine	2%

Most of the data on teratogenicity of AEDs are derived from observational studies as randomized clinical trials have no place on human subjects [12]. In data collected from the UK Epilepsy & Pregnancy Register on 3,607 cases, MCM rates among fetuses exposed in utero to AEDs was 4.2%, and the risk was also higher for those exposed to polytherapy (6.0%) as compared to monotherapy (3.7%). The MCM rate in WWE not exposed to AEDs during pregnancy was 3.5%. The MCM rate was 6.2% for valproate, 2.2% for carbamazepine, and 3.7% for phenytoin (Table 5). Positive dose and malformation rates were observed for lamotrigine [13].

**Table 5:** UK Epilepsy & Pregnancy Register and Malformation Rates with AEDs AED, anti-epileptic drug; MCM, major congenital malformation.

AED	MCM Rates
Valproate	6.2%
Phenytoin	3.7%
Carbamazepine	2.2%

^The specific type of malformations associated with a particular type of AED^

Results from data of the EURAP, NAAPR, and UK Epilepsy & Pregnancy Register showed that valproate is associated with the highest rates of abnormalities, whereas carbamazepine and Lamotrigine are associated with moderately increased risk. [9,11,13] In this section, we will discuss briefly individual AED and types of malformation associated with each drug.

^Valproate^

The NAAPR results showed that valproate is associated with an MCM rate of 9.3%, whereas the UK Epilepsy & Pregnancy Register reported an MCM rate of 6.2%. [11,13]. Use of valproate during gestation can lead to neural tube defects frequently complicated by hydrocephaly and other midline neural defects [14]. 

^Phenytoin^

The NAAPR and the UK Epilepsy & Pregnancy Register showed that malformation rates with phenytoin are 2.9% and 3.7%, respectively [11,13]. It can cause an increased risk of neural tube, orofacial, cardiovascular, and genitourinary tract defects [10]. 

^Phenobarbital^

The NAAPR reported that the malformation rate of phenobarbital is 5.5% [11]. Since it is a folic acid antagonist, it causes neural tube, cardiovascular, orofacial, and genitourinary malformations [10]. 

^Carbamazepine^

The NAAPR reported MCM rates for carbamazepine to be 2.9%, whereas the UK Epilepsy & Pregnancy Register reported it to be 2.2% [11,13]. Carbamazepine is widely available and the most commonly used AED in WWE. A research study conducted by Ornoy and Cohen showed development delay in children born to WWE exposed to carbamazepine during pregnancy. A total of 47 children were assessed by a pediatrician with complete physical and neurodevelopment assessment, of whom six had carbamazepine syndrome in group A with typical facial dysmorphic features of up-slanting palpebral fissures, epicanthic folds, micrognathia, broad nasal bridge, high arched palate/cleft palate, and mild mental retardation. About one-third of the women in the group had seizures in pregnancy, but there was no correlation between maternal seizures, facial dysmorphism, and mental retardation. However, one possible explanation for the occurrence of carbamazepine syndrome was the difference in the genetics of enzyme epoxide hydrolase between populations, which might be responsible for carbamazepine-induced embryopathy [15]. 

^Lamotrigine^

The NAAPR reported malformation rates for lamotrigine to be 2% (11). The UK Epilepsy & Pregnancy Register reported fewer malformation rates with lamotrigine, but positive dose-response and malformation rates were observed [13]. In the NAAPR infants exposed to lamotrigine, 16 out of 684 had major malformations, with 5 having oral clefts either isolate cleft lip, cleft palate, or both cleft lip and palate [16].

^Levetiracetam^

The UK Epilepsy & Pregnancy Register and the Irish Epilepsy and Pregnancy Register collected observational data of WWE exposed to levetiracetam during the first trimester of pregnancy. From the data of 671 pregnancies with 304 exposed to monotherapy and 367 exposed to polytherapy, only two cases of MCM in the monotherapy group (0.7%) and 19 cases in the polytherapy group (6.47%) were observed. MCM varied with the regimen used in polytherapy with levetiracetam. Rates were lower when combined with lamotrigine (1.77%) than with valproate (6.9%) and carbamazepine (9.38%). Levetiracetam is generally considered a safe option for women of childbearing age as a treatment regimen [17].

^Oxcarbazepine^

Few data are available on this newer generation of AEDs, but they are generally considered safe, with no malformation rates reported. In one case report, a 23-year-old female with a history of complex partial seizure and mild depression was initiated on a combination therapy of phenytoin and gabapentin for several years. After planned pregnancy, the patient was switched to oxcarbazepine, and both phenytoin and gabapentin were tapered off. The patient was maintained on monotherapy and delivered a healthy baby [18]. However, there is very limited data on newer AEDs such as oxcarbazepine safety in pregnancy; it needs more studies for its consideration as a safe option for management during pregnancy.

^Comorbidities, complications, and health issues in WWE^

Epilepsy also increases the risk of other psychiatric disorders two to three times more as compared to the general population, such as mood disorder, anxiety, depression, and suicidal risk [19]. These conditions need to be assessed and screened carefully by the obstetrician during pregnancy. Upon diagnosis, it needs to be managed along with a psychiatrist to improve the well-being and outcome in pregnant WWE. Exposure to AED also increases the risk of osteopenia and osteoporosis due to alteration in bone metabolism. Increase in the fracture risk is due to seizures and exposure to AED. It is important to prescribe patients taking AED with prophylactic calcium and vitamin D and to counsel on good bone health practices [20]. 

^Long-term effects of AEDs on neurocognition, psychology, and behavioral development in offspring^

Numerous observational studies have been conducted to determine the effect of in utero exposure to AEDs on neurocognition and behavioral outcome of offspring. One Danish-based cohort study was conducted by Bech et al. to determine the association between exposure to AEDs and learning disabilities in the children that include mental retardation, autism spectrum disorder, emotional, behavioral disorders and children’s requiring special educational needs in the first year of life. A study conducted between 2005 and 2011 included 117,475 live births, with 636 cases and 434 controls. Learning disabilities were found in 7.1% of infants exposed to AEDs in utero as compared to 3.7% of the controls. Valproate is associated with a significantly increased risk of learning disabilities, with an OR of 4.67%, and lamotrigine is associated with the lowest risk, with an OR of 0.42% [21]. 

Hence, with reference to other observational studies as well, valproate must be avoided for seizure control in WWE of childbearing age to avoid malformations in fetus development and to prevent learning disabilities.

Another retrospective study was conducted in regional epilepsy clinics in Manchester and Liverpool in the UK to study the long-term outcome of children born to mothers with epilepsy. The study included 249 children aged six and older in the survey, with 41 exposed in utero to sodium valproates, 52 to carbamazepine, 21 to phenytoin, and 49 to polytherapy AEDs, and 80 were non-exposed. The study results found that exposure to valproate in children is associated with increased risks of developmental and cognitive delays. Tonic-clonic seizures in pregnancy were also associated with low verbal intelligence quotient in children even after adjusting other confounding factors [22]. WWE need careful counseling before conception about risks and benefits associated with AED treatment during pregnancy.

^Breastfeeding and AEDs^

Breastfeeding is encouraged in WWE. The amount of AEDs transferred through breast milk is in smaller quantities as compared to the levels exposed in utero. But newborns do not have a fully developed drug-eliminating mechanism; hence, there can be an accumulation of antiepileptic medications [23]. 

^Recommendations for the optimal management of epilepsy in pregnancy^

Most of the data on teratogenicity and malformation rates due to AEDs are collected from observational studies as clinical trials cannot be conducted. We only have level B and lower recommendations. Some important recommendations for WWE are as follows:

(1) It is essential to supplement with 0.4 mg of folic acids in WWE before conception to reduce the risk of MCM (level C) [24].

(2) Lamotrigine, carbamazepine, and phenytoin levels change significantly during pregnancy due to their increased clearance and can lead to an increase in the seizure frequency. Monitoring of these medicines should be considered (level B) [24].

(3) Two class III studies have shown a decrease in the active metabolite of oxcarbazepine. One class II study has also shown a decrease in the plasma concentration of levetiracetam by 60% in the third trimester as compared to preconception baseline line levels. Hence, monitoring of plasma levels of oxcarbazepine and levetiracetam might be considered (level C) [24].

(4) Valproate is associated with the highest risks of malformation rates and should never be considered the first line of treatment for WWE considering pregnancy. Carbamazepine and lamotrigine seem comparatively safer options with twofold higher risks than controls, but lamotrigine is associated with the pharmacokinetics changes in drug levels during pregnancy and can lead to breakthrough seizures. Carbamazepine is the first choice of drug in localized epilepsy [25]. 

(5) Most WWE can conceive and have normal healthy children with the optimal management approach, but they are at an increased risk of developing complications due to which they need to be monitored continuously during pregnancy [26]. 

(6) Guidelines also recommend optimizing treatment before conception, selecting the most effective AED for specific seizure type and syndrome, using the lowest effective dose on monotherapy, and supplementing with folate and multivitamins [12]. 

(7) Despite the small increase in the risk of fetal malformations associated with AEDs, women should be encouraged to strictly adhere to treatment during pregnancy. It is important to rule out any anatomical abnormality through high-resolution anatomical scans performed at 18-20 weeks [23]. 

(8) Most of the WWE generally have a vaginal delivery, and the mode of delivery can be decided according to obstetric indications rather than epilepsy itself [27].

## Conclusions

Epilepsy requires lifelong intake of AEDs. Most of the research regarding the teratogenicity of AEDs are derived from observational studies. Epilepsy affects every phase of reproductive and general health of women. It needs to be dealt with constant emotional and psychological support and counseling from physicians, obstetricians, and family members. More advancement and research are required to develop a permanent cure for this chronic neurological illness, such as stem cell therapy and surgical resection of foci of the brain.
